# Synthesis, characterisation, and *in vitro* assessment of hyaluronic acid-coated solid lipid nanoparticles entrapping dexamethasone palmitate designed for intra-articular administration

**DOI:** 10.3389/fddev.2026.1809275

**Published:** 2026-09-04

**Authors:** Jinxin Zhang, Rumi Khandelia, Bridget Hogg, Ahmad Z. Bahlool, Tom Hodgkinson, Sally-Ann Cryan, John O’Byrne, David J. Brayden

**Affiliations:** 1 UCD School of Veterinary Medicine, University College Dublin, Dublin, Ireland; 2 UCD Conway Institute, University College Dublin, Dublin, Ireland; 3 Department of Pharmacy and Biomolecular Science, Royal College of Surgeons in Ireland, Dublin, Ireland; 4 Department of Pharmacy, Faculty of Pharmacy, Al-Zaytoonah University of Jordan, Amman, Jordan; 5 Department of Anatomy and Regenerative Medicine, Royal College of Surgeons in Ireland, Dublin, Ireland; 6 National Orthopaedics Hospital Cappagh, Dublin, Ireland

**Keywords:** corticosteroids, hyaluronic acid, intra-articular administration, knee osteoarthritis, microfluidics, solid lipid nanoparticles

## Abstract

Osteoarthritis (OA) is the major cause of pain and disability worldwide, particularly affecting the knee joint. Although intra-articular (IA) treatments including corticosteroids and hyaluronic acid (HA), are commonly used to manage symptoms, their therapeutic effects are often short-lived, necessitating repeated injections. In this study, HA-coated solid lipid nanoparticles (HA-DXP-SLNs) entrapping with the glucocorticoid prodrug, dexamethasone palmitate (DXP) was developed as dual-function nanocarrier system for IA delivery. Using a scalable microfluidics platform, monodisperse SLNs of ∼90 nm in diameter were produced with high DXP encapsulation efficiency (88%) and loading capacity (6% w/w). In phosphate buffered saline (PBS), DXP-SLNs exhibited a biphasic release profile with an initial burst followed by sustained release of DXP, which was further moderated by HA surface coating. HA coatings of the SLNs enhanced particle stability, shifted particle surface charge from positive to negative, and enabled uptake by patient-derived primary osteoarthritic chondrocytes (hOAAC). Incubation in murine plasma demonstrated enzymatic conversion of DXP to dexamethasone (DEX), which accumulated in plasma over 72 h. HA-DXP-SLNs suppressed prostaglandin E_2_ production by LPS-stimulated THP-1 monocytes and by hOAAC more effectively than uncoated DXP-SLNs, while maintaining cell viability. These findings indicate that the HA coating of SLNs contributes to *in vitro* therapeutic efficacy of the overall SLNs, as well as to particle stabilisation. HA-DXP-SLNs therefore represent a promising nanocarrier for prolonged IA corticosteroid delivery in knee OA, with the potential to reduce the frequency of current IA corticosteroid and HA administration.

## 1 Introduction

Osteoarthritis (OA) of the knee is the most common arthritis and a leading cause of disability, impairing daily function and quality of life. One in eight adults aged over the age of 60 has knee OA (O’Neill and Felson, 2018). In 2020, OA affected approximately 595 million people worldwide, representing 7.6% of the global population, and remains one of the leading causes of disability among older adults. The global burden of OA continues to increase due to population ageing and growth, with case numbers projected to rise substantially by 2050, particularly for knee OA. (Hunter et al., 2020; Cui et al., 2020; Steinmetz et al., 2023; Cao et al., 2024). OA is now recognised as a disease that affects the entire joint, rather than being limited to the degeneration of articular cartilage alone. The condition is characterised by progressive cartilage breakdown, synovial inflammation, subchondral bone remodelling, and alterations in inflammatory signalling across multiple joint tissues. While OA has traditionally been viewed as a degenerative disease, growing evidence indicates that low-grade inflammation plays an important role in its progression.

The inflammatory response can activate chondrocytes, synoviocytes, and immune cells within the joint, contributing to tissue damage and disease development (van den Bosch, 2021). Cartilage breakdown caused by proteolytic enzymes drives OA. Aggrecanases (ADAMTS-4, ADAMTS-5) and collagenases (matrix metalloproteinase-1 (MMP-1, MMP-8, MMP-13) degrade proteoglycans and type II collagen, disrupting the balance between cartilage degradation and repair (Abramson and Attur, 2009). Although anabolic factors including IGF-I, TGF-β, BMPs, and FGFs promote cartilage repair, Pro-inflammatory cytokines such as IL-1β and TNF-α play a central role in driving cartilage degradation in OA by promoting a catabolic phenotype in chondrocytes. This results in increased production of matrix-degrading enzymes, as well as inflammatory mediators including nitric oxide (NO) and prostaglandin E_2_ (PGE_2_). Elevated levels of PGE_2_ have been associated with reduced extracellular matrix synthesis and enhanced catabolic activity, whereas NO contributes to chondrocyte dysfunction and further degradation of cartilage matrix components. As these catabolic mechanisms become dominant over reparative processes, the balance within the joint shifts towards progressive cartilage loss and disease progression (van den Bosch, 2021).

Knee OA treatment focuses on pain management and slowing disease progression through a combination of non-pharmacological, pharmacological, and surgical approaches. Non-pharmacological interventions include weight management, exercise, acupuncture, and supportive footwear, while pharmacological therapies may be administered *via* oral or IA routes. Current management of OA is therefore primarily aimed at alleviating symptoms through these combined strategies. Among pharmacological options, IA corticosteroid injections are commonly used for the treatment of symptomatic knee OA and are recommended in current clinical guidelines. However, the benefits of these injections are often short-lived, with symptom relief typically diminishing over time, resulting in the need for repeated administration to maintain therapeutic efficacy (Anandacoomarasamy and March 2010; McCrae et al., 2018; Kolasinski et al., 2020). Paracetamol is commonly used as a first-line oral analgesic for OA, however, its effects on pain and function are modest, with limited improvement in WOMAC outcomes reported in some patient populations. Oral non-steroidal anti-inflammatory drugs (NSAIDs) remain among the most effective pharmacological options for symptomatic OA management through inhibition of cyclooxygenase (COX) enzymes and subsequent prostaglandin synthesis. Despite their clinical efficacy, long term NSAID use is associated with an increased risk of gastrointestinal, renal, and cardiovascular adverse events, particularly in older adults and patients with existing comorbidities, which may limit their prolonged use. In contrast to oral administration, IA injections offer potential for high local drug concentration with reduced systemic side effects. FDA-approved IA therapeutic options for knee OA however are currently limited to analogues of glucocorticoids and hyaluronic acid (HA) (Cao et al., 2021; Wehling et al., 2017).

Glucocorticoids act by suppressing cartilage production of inflammatory mediators (leukotrienes, prostaglandins) and inhibiting neutrophils and metalloproteases, reducing swelling, tenderness, and by improving viscosity through boosting HA levels in the joint. In 2019, the OA Research Society International and the European Society for Clinical and Economic Aspects of Osteoporosis and OA recommended IA-administered glucocorticoids as a treatment for knee OA (Mountziaris et al., 2009). On particular agent, dexamethasone palmitate (DXP), is a lipophilic prodrug of the potent glucocorticoid, dexamethasone (DEX). DXP comprises a C16 palmitic ester chain to form hydrophobic interactions. In synovial fluid of the knee, esterases can convert DXP to active DEX using local esterases, allowing sustained release and extended activity for up to 3 weeks (Spitzer et al., 2019). This is the rationale for including DXP as a preferred glucocorticoid in an IA particulate formulation (Mountziaris et al., 2009; Spitzer et al., 2019).

HA, a glycosaminoglycan present in synovial fluid, acts as a joint lubricant and shock absorber. It binds to Cluster of Differentiation 44 (CD44) and layilin receptors on chondrocytes and exerts independent anti-inflammatory effects. It regulates MMP-13 in subchondral bone and forms the backbone of proteoglycans in the extracellular matrix. (Craciunescu et al., 2021; Ryan et al., 2013). CD44 is a principal cell-surface receptor for HA and is involved in cell adhesion, migration, inflammatory signalling, and extracellular matrix cell interactions. In OA, altered HA–CD44 interactions have been associated with chondrocyte activation, cartilage degradation, and inflammatory responses (Saniya Salathia, 2023). Kang et al. (2021) demonstrated increased CD44 expression in damaged cartilage from OA patients and OA mouse models, and showed that CD44 activation increased MMP3, MMP13, COX2, collagenase activity, as well as PGE_2_ production in chondrocytes. Their study further demonstrated that HA nanoparticles attenuated IL-1β- and CD44 induced catabolic responses in chondrocytes, supporting the relevance of HA–CD44 interactions in OA-associated inflammation and cartilage degeneration (Kang et al., 2021).

HA-functionalised nanosystems have also been widely investigated for CD44-mediated targeting of inflammatory cells and arthritic tissues due to the affinity of HA for CD44-overexpressing cells. CD44 expression has been reported on activated macrophages, synoviocytes, and chondrocytes within inflamed joint tissues, making HA an attractive surface ligand for targeted nanoparticle delivery in inflammatory joint diseases. Therefore, HA coating in the present study was used not only to improve nanoparticle stability and surface properties, but also to provide a biologically relevant HA surface potentially capable of interacting with CD44 expressing cells within the OA microenvironment in addition to its known independent anti-inflammatory action (Saniya Salathia, 2023) Priya et al. (2025) also provided evidence for the use of HA as a CD44 targeting surface ligand in inflammatory arthritis. In their study, HA coated lyotropic liquid crystalline nanoparticles were designed to target CD44 overexpressing inflammatory cells, particularly activated macrophages and fibroblast-like synoviocytes in arthritic tissue. They reported that HA-coated nanoparticles showed higher cellular uptake than uncoated nanoparticles and that HA-functionalised nanoparticles reduced inflammatory cytokines and CD44 expression in an arthritic rat model. This supports the rationale that HA coating can provide both surface stabilisation and a CD44 interactive interface for delivery to inflamed joint-associated cells. Since CD44-mediated uptake of HA is well known, it was not further investigated in the present study.

Previous studies in rheumatoid arthritis (RA) have demonstrated the potential of HA-functionalised nanocarriers for targeted drug delivery within inflamed joints. HA-coated nanoparticles have been shown to interact with CD44-expressing cells, including macrophages and fibroblast-like synoviocytes, leading to enhanced accumulation within diseased joint tissues (Zewail et al., 2022; Priya et al., 2025). Although RA and OA are distinct disease entities, these findings provide supporting evidence for the use of HA as a targeting ligand in OA, where CD44 is also expressed by chondrocytes and other resident joint cells.

In OA, the synovial fluid becomes less viscous due to a decrease in the molecular weight of HA. IA injections of HA restore viscoelasticity, reduce cytokines (TNF-α, IL-1), and promote cell repair. Several HA-formulations are FDA-approved for IA-injection to treat knee OA (Bhandari et al., 2017; Cao et al., 2021; Ayhan et al., 2014). However, HA and steroid IA injections are limited by rapid clearance and poor penetration of the dense, negatively charged extracellular matrix. IA-injected steroids typically remain in knee joints for 1–4 h, while HA lasts only ∼26 h. Drug diffusion within the knee joint is hindered by high synovial fluid viscosity and cartilage structure. Repeated IA injections may cause swelling, infection, and increase septic arthritis risks (Sladek et al., 2018; Brown et al., 2019), hence new therapeutic IA formulations are needed to promote joint retention and lasting benefits beyond a few weeks.

Nanoparticles (NPs) may improve IA delivery to the arthritic knee by prolonging retention, enhancing cartilage penetration, and reducing off-target effects. They can be passively targeted using cationic NPs to bind to negatively-charged cartilage or actively targeted with ligands including collagen type II antibodies (Brown et al., 2019; Li et al., 2021). However, NPs penetration to knee cartilage is size dependent and they can struggle to pass through the dense superficial cartilage matrix, which has a mesh size of ∼50–60 nm and proteoglycan spacing of ∼20 nm (Li et al., 2021). Encapsulating DXP in NPs can potentially prolong release, improve targeting, and may lower side effects associated with free glucocorticoids. NP accumulation in knee cartilage is further aided by the inflammation-driven vascular leakage and immune uptake effect (Tessier et al., 2023). Combining DXP-entrapped NPs with surface coated HA could therefore enable CD44-mediated targeting of chondrocytes, in addition to the inherent benefits of the two agents.

In terms of NP selection for IA administration, solid lipid nanoparticles (SLNs) are an attractive option. With diameters that can be engineered to be in the 50–500 nm range, they are biocompatible, stable, and suitable for both hydrophilic and hydrophobic drug entrapment, thereby allowing controlled release and scalable production (Khairnar et al., 2022). However, conventional SLN synthesis faces variability and scalability issues. Microfluidic methods overcome these by ensuring uniform particle size, higher encapsulation efficiency, and continuous, reproducible production, as demonstrated for mRNA Covid vaccines (Tenchov et al., 2021). Using SLNs as delivery systems for IA administration to knee joints may approach offer effective IA therapies with few side effects (Shepherd et al., 2021; Vogelaar et al., 2023). DXP has been approved for IA administration for humans as a liposome formulation, Lipotalon®, Ricordati, Italy), but it has been discontinued (Mountziaris et al., 2009). This creates an opportunity for using SLNs for IA administration of DXP with a HA coating. Aside from liposomes, a microparticle formulation, Zilretta® (triamcinolone acetonide extended-release, Pacira Biosciences, NJ, United States), was FDA-approved for IA administration to the knees of OA patients in 2017 (Spitzer et al., 2019). It delivers 32 mg triamcinolone acetonide in poly(lactide)-co-glycolide (PLGA) microspheres over 3 months. The initial burst release associated with PLGA microspheres underscores the need for stable IA therapies using particle constructs with improved control of release (Paik et al., 2019). A Phase III trial in 2019 returned positive safety and efficacy data for Zilretta® for a second IA administration with flexible dosing regimens (Spitzer et al., 2019), however it is not yet FDA-approved for repeat administration. There remains a gap in therapy for other types of NP with varieties of payloads.

Our overall hypothesis was that the combination of DXP and HA in SLNs can eventually provide a durable anti-inflammatory effect for knee OA when administered by IA injection and which will be superior to current stand-alone steroid and HA injections. In this initial study, the first aim was to generate a reproducible synthesis protocol for HA-DXP-SLNs that is amenable to scalable microfluidic production. The second aim was to provide evidence of an anti-inflammatory effect of the multifunctional SLNs in primary cells from patients, along with acceptable biocompatibility.

## 2 Materials and methods

### 2.1 Chemicals

DXP, tristearin, 1,2-dioleoyl-3-trimethylammonium-propane (DOTAP), 1,2-dimyristoyl-rac-glycero-3-methoxypolyethylene glycol-2000 (DMG-PEG 2000), and HA (the sodium salt from *Streptococcus* equi, MW 150,000–300,000) were obtained from Sigma-Aldrich (Ireland). Amicon® Ultra centrifugal filters (3 kDa MWCO and 300 kDa MWCO), Microcon® centrifugal filters, and Biomax® 300 kDa membranes were purchased from Merck (Ireland). Roswell Park Memorial Institute (RPMI 1640) medium, Dulbecco’s Modified Eagle’s Medium (DMEM; low glucose), and DMEM/Nutrient Mixture F-12 Ham were supplied by Sigma-Aldrich. Lipopolysaccharide (LPS) from *Escherichia coli* O55:B5 (γ-irradiated, BioXtra grade, suitable for cell culture) and DEX powder were also obtained from Sigma-Aldrich.

Pronase from *Streptomyces griseus*, fibroblast growth factor 2, trypsin-EDTA solution, sodium pyruvate (100 mM), L-ascorbic acid 2-phosphate sesquimagnesium salt hydrate, PBS tablets, sodium bicarbonate, MTS tetrazolium dye, and Triton X-100 were all purchased from Sigma-Aldrich. RPMI medium 1640 (1×) supplemented with GlutaMAX-1, foetal bovine serum, penicillin-streptomycin (10,000 U/mL), MEM non-essential amino acids (100×), and insulin–transferrin–selenium–ethanolamine (100×) was supplied by Gibco (Thermo Fisher Scientific, United States). Opti-MEM™ I reduced-serum medium was obtained from Gibco.

The PGE_2_ ELISA kit (monoclonal) and ultrapure water were purchased from Cayman Chemical Company. The hyaluronic acid ELISA kit (HA/Hyaluronic Acid ELISA Kit, UNFI0033) was purchased from Assay Genie, Ireland. The CellTiter 96® AQueous One Solution Cell Proliferation Assay (MTS) was obtained from Promega. HPLC-grade CHROMASOLV™ Plus water was purchased from Honeywell. Alexa Fluor® 568 NHS ester, Hoechst 33,342 (trihydrochloride, trihydrate; 10 mg/mL in water), and Alexa Fluor® 488 phalloidin were purchased from Invitrogen. Formaldehyde fixative (16% w/v, ultrapure EM grade; MaxTag Histo for IHC) was obtained from Rockland Immunochemicals. Unless otherwise stated, all chemicals were used as received without further purification.

### 2.2 Synthesis of SLNs using manual hot homogenisation

Control and steroid-loaded SLNs were prepared manually by hot homogenisation (HH). Tristearin (2%–2.5% w/v) was melted at 70 °C, and DEX (used initially instead of DXP to establish the principle) was incorporated at selected drug-to-lipid ratios together with DOTAP (0.5% w/v) to form the organic phase. The aqueous phase contained Tween® 80 (0.5% w/v) and was preheated to 70 °C. It was added dropwise to the organic phase and homogenised at 4074 g (Ultra Turrax T23, IKA-Werke GmbH, Germany) for 10 min at 70 °C. The hot emulsion was cooled to room temperature, and residual solvent was removed using a rotary evaporator. NPs were purified by ultracentrifugation (50,000 g, 60 min) with additional washing, and the pellet was resuspended in water for characterisation. In HH studies, HA coating was not performed.

### 2.3 Synthesis of SLNs using the Ignite® microfluidics system

**FIGURE 1:**
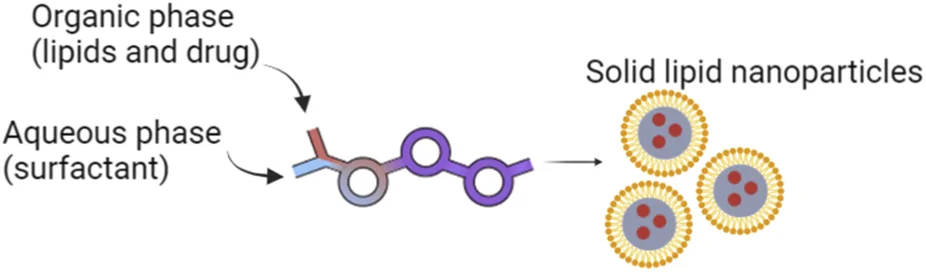
Formation of SLN using NanoAssemblr™ Ignite™ system.

SLNs were prepared using the NanoAssemblr™ Ignite™ system (Precision Nano-Systems Inc., Vancouver, Canada). The system enables precise control of total flow rate (TFR) and flow rate ratio (FRR) to achieve uniform mixing. A lipid-to-aqueous phase ratio of 1:3 was selected based on data from the manual process. Tristearin (20 mg/mL in absolute ethanol), DOTAP (20 mg/mL in DMSO), and DXP (25 mg/mL in DMSO) were dissolved in ethanol at 70 °C (organic phase). The aqueous phase contained DMG-PEG 2000 (10 mg/mL). The two phases were introduced into the Ingite® NxGen™ cartridge *via* separate inlets (Figure 1) using predefined flow rates and injection volumes. The phases merged within the cartridge and exited as an SLN suspension. Formulations included control, DEX/DXP-entrapped, and rhodamine-123–labelled SLNs. The suspension was collected in 15 mL tubes and purified by ultrafiltration–centrifugation (Amicon Ultra-15, MWCO 50 kDa; Millipore, United States) for 40 min at 4500 g.

### 2.4 Synthesis of HA-coated drug-loaded SLNs from the Ignite® microfluidics system

Purified DXP-SLNs were mixed with HA (3% w/v) in a 2 mL vessel under magnetic stirring at room temperature for 3 h. HA-DXP-SLNs were purified by centrifugation (Microcon® centrifugal filters MWCO 30 kDa) at 5,000 g for 10 min at 4 °C. The stability of control SLNs and HA-DXP-SLNs was assessed over 4 weeks at 4 °C.

### 2.5 Morphology of SLNs made by HH and microfluidics

The hydrodynamic diameter, polydispersity index (PDI) and zeta potential of SLNs were determined by dynamic light scattering (DLS) using a Nano Zetasizer, Malvern Instruments, UK). Prior to analysis samples were diluted in a ratio of 10:990 in ultrapure water and allowed to equilibrate for 2 min at 25 °C. Measurements were carried out at a scattering angle of 173° using disposable cuvettes. Each sample was measured using 10 to 15 runs, with all measurements performed in triplicate. Morphology was assessed by transmission electron microscopy (TEM). Diluted suspensions of DXP-SLNs and HA-DXP-SLNs were placed on carbon-coated copper grids, dried overnight at room temperature, and imaged using an FEI Tecnai G2 12 BioTWIN TEM at 120 kV.

### 2.6 Encapsulation efficiency of SLNs made by HH and microfluidics

The encapsulation efficiency (EE%) and drug loading (DL%) of DEX and DXP from SLN produced by HH and microfluidics were quantified by HPLC (Agilent 1200 Series, Agilent Technologies, Cork, Ireland). For DEX-SLNs, isocratic elution was performed with a 50:50 phosphate buffer (pH 6.8 at room temperature) with an acetonitrile mobile phase at 1 mL/min, using a Symmetry C18 column (250 × 4.6 mm, 5 μm; Waters, Wexford, Ireland). Detection of DEX was at 241 nm with a 10 µL injection volume with retention time of 3 min. Calibration curves (1.56–100 μg/mL) yielded *R* ^2^ = 0.99. For HA-DXP-SLNs, isocratic elution used 15% water:85% acetonitrile at 1.2 mL/min, with the same column maintained at 40 °C. Detection of DXP was at 240 nm with a 10 µL injection volume with retention time of 27 min. Calibration curves for DXP (1.56–100 μg/mL) also yielded *R* ^2^ = 0.99.

Because DXP is highly lipophilic and poorly soluble in aqueous media, samples were not analysed directly in aqueous suspension. Prior to HPLC analysis, all DXP-SLNs and HA-DXP-SLNs were subjected to an organic-solvent extraction/solubilization step. Briefly, 10 µL of sample was mixed with isopropanol buffer (10 mM, 1:1), sonicated for 1 h to disrupt the lipid nanoparticles and extract/solubilize DXP, and centrifuged at 1000 *g* for 10 min. The resulting supernatant was transferred to HPLC vials (Thermo Fisher Scientific) for analysis.

EE % and DL % for DEX and DXP from both HH and the Ignite® microfluidics system were calculated using the following equations:

$$\text{EE }\%=\left(W_{\text{total}-}W_{\text{free}}\right)/W_{\text{total}}\times 100$$

$$\text{DL }\%=\left(W_{\text{total}}-W_{\text{free}}\right)/W_{\text{lipid}}\times 100$$

Where W_total_: total weight of drug added in the formulation

W_free_: free drug

W_lipid_: total weight of lipid used in the formulation

### 2.7 Quantification of HA in HA-DXP-SLNs made by microfluidics

HA content on coated SLNs was determined by ELISA according to the manufacturer’s protocol. Samples were diluted in PBS, and a standard curve was generated in PBS.

### 2.8 Attenuated total reflectance-fourier transform Infrared spectroscopy (ATR-FTIR) analysis for HA-DXP-SLNs

500 µL of HA, DXP-SLNs, and HA-DXP-SLNs made by microfluidics were aliquoted into 1.5 mL Eppendorf tubes, sealed with Parafilm™ (perforated for air exchange), and frozen at −20 °C for 2 h before being lyophilised for 72 h at a temperature of −55 °C and pressure of 0.023 mbar using a LyoStar™ 3 freeze-dryer (Labconco, United States). Thermocouples (36-gauge, Type T; Omega Engineering, United States) were inserted into representative vials to monitor product temperature during the process. Following lyophilisation, ATR-FTIR spectroscopy was performed to characterise potential chemical interactions between HA, DXP-SLNs, and HA-DXP-SLNs. Dried samples were placed directly onto the ATR crystal of a Fourier transform infrared spectrometer equipped with an attenuated total reflectance sampling accessory, ensuring full contact between the sample and the crystal surface. Spectra were collected over the range of 4000 to 0 cm^−1^ at a spectral resolution of 2 cm^−1^, averaging 16 scans per sample to enhance the signal-to-noise ratio. The resulting spectra for HA, uncoated DXP-SLNs, and HA-DXP-SLNs were compared to identify characteristic functional groups and to confirm the presence of HA on the nanoparticle surface, following the approach outlined in the referenced protocol for HA-coated SLNs.

### 2.9 *In vitro* release of DXP from DXP-SLNs and HA-DXP-SLNs

*In vitro* release of DXP from free DXP, DXP-SLNs (made by microfluidics), and HA-DXP-SLNs (made by microfluidics and then coated with HA) were studied by dialysis. Aliquots (2 mL) of each formulation were transferred into dialysis bags with a molecular weight cut off of 3.5 kDa. The sealed dialysis bags were immersed in 50 mL of phosphate-buffered saline (PBS, pH 7.4) and maintained at 37 °C. At predetermined time intervals for up to 120 h, 1 mL samples of the release medium were withdrawn and immediately replaced with an equal volume of fresh buffer. The collected samples were transferred to HPLC vials (Thermo Fisher Scientific) for determination of the cumulative amount of DXP released overtime as described in Section 2.6.

### 2.10 *In vitro* conversion to DEX from DXP-SLNs and HA-DXP-SLNs in mouse plasma

DXP-SLNs made by microfluidics were diluted in water to a final DXP concentration of 260 μg/mL. Murine plasma (180 µL) was mixed with 20 µL of DXP-SLNs and incubated at 37 °C for up to 72 h. At each time point, samples were collected into separate tubes. Acetonitrile (400 µL) containing DEX acetate (4 μg/mL, internal standard) was added, vortexed for 30 s to precipitate plasma proteins, and centrifuged at 4696 *g* for 10 min (ST16R centrifuge, TX-400 rotor; Thermo Scientific, Ireland). The supernatant was transferred to HPLC vials (Thermo Fisher Scientific) for analysis as described in Section 2.5 (Lorscheider et al., 2019).

### 2.11 Effects SLNs on the cellular viability of THP-1 monocytes: MTS assay

Human THP-1 monocyte cells were seeded at 1 × 10^5^ cells/well in 96-well plates with serum free RPMI 1640 media and allowed to adhere for 30 min. Cells were then exposed to DXP-SLNs (at DXP equivalent concentrations of 0.125 µM, 0.25 µM, 0.5 µM, and 0.75 µM) or HA-DXP-SLNs (at DXP equivalent concentrations of 0.125 µM, 0.25 µM, 0.5 µM, and 0.75 µM). Free DXP (0.5 µM) and HA alone (3 mg/mL) were included as controls Triton® X-100 (0.1% v/v) served as a positive control. The final volume in each well was 150 µL.

After 24 h incubation with test agents, plates were centrifuged at400 *g* for 5 min at room temperature and the supernatants were removed. MTS assay was performed. Briefly, MTS reagent was added to the required wells, then the plate was incubated for 3 hours. at 37 °C. Absorbance was then read at 490 nm. Each condition was evaluated in triplicate across two independent experiments.

### 2.12 Effects of HA-DXP-SLNs on PGE_2_ secretion from lipopolysaccharide (LPS)-stimulated THP-1 monocytes

THP-1 cells (1 × 10^5^/well) were seeded in 96-well plates in serum free RPMI 1640 media and incubated with control-SLNs (at final DXP equivalent concentration of 0.5 µM), DXP-SLNs (at final DXP equivalent concentration of 0.125 µM, 0.25 µM, and 0.5 µM) and HA-DXP-SLNs at final DXP equivalent concentration of 0.125 µM, 0.25 µM, and 0.5 µMat for 2 h. Free DXP (0.5 µM) and HA (3 mg/mL) were included as controls. Following the 2 h incubation period, LPS (1 μg/mL) was added directly to each required well and cells were incubated for a further 24 h at 37 °C in a humidified 5% CO_2_ incubator. The final volume in each well was 150 µL.

After incubation, plates were centrifuged at 400 *g* for 10 min at 4 °C. Conditioned media were collected and stored at −80 °C prior to analysis. PGE_2_ concentration were quantified by a commercial ELISA kit according to the manufacturer’s protocol using undiluted samples. Standard curves were prepared in serum-free media RPMI 1640 medium. Data were analysed with a four-parameter logistic (4 PL) curve fit in GraphPad® Prism 8. Each condition was assessed in three independent biological experiments.

### 2.13 Effects of HA-DXP-SLNs on the cellular viability of hOAAC: MTS assay

Primary human osteoarthritic articular chondrocytes (hOAAC) were seeded at 5 × 10^4^ cells/mL in 96-well plates and cultured overnight in expansion media at 37 °C in a humidified 5% CO_2_ incubator. Cells were washed twice with PBS and treated in Opti-MEM™ (0.1% FBS) with free DXP (0.5 µM), HA-DXP-SLNs made by microfluidics (at final DXP equivalent concentrations of 0.125 µM, 0.25 µM, and 0.5 µM), control SLNs (at a final DXP equivalent concentration of 0.5 µM)), or Triton X-100 (0.1% v/v). The final volume in each well was 150 µL. After 24 h, media were removed, cells washed twice with PBS, and viability assessed by MTS assay. Briefly, MTS reagent was added to wells, then the plate was incubated for 3 hours. at 37 °C. Absorbance was then read at 490 nm to obtain the results. Experiments were performed with hOAAC from three human donors.

### 2.14 Effects of HA-DXP-SLNs on secreted PGE_2_ in LPS-stimulated hOAAC

hOAAC (5 × 10^4^ cells/mL) were seeded in 96-well plates and cultured overnight at 37 °C in a humidified 5% CO_2_ incubator. After washing twice with PBS, cells were treated in Opti-MEM™ (0.1% FBS) with free DXP (0.25 µM), HA-DXP-SLNs at final DXP equivalent concentrations of 0.125 µM, 0.25 µM, 0.5 µM, control SLNs (at final DXP equivalent concentration of 0.5 µM) Free Dex (0.5 µM, free DXP (0.5 µM), and HA (0.45 mg/mL) were included as controls. The final volume in each well was 150 µL.

After 24 h, conditioned media were collected and stored at −80 °C prior to analysis. PGE_2_ concentration were quantified by a commercial ELISA kit according to the manufacturer’s protocol using diluted samples. Standard curves were prepared in Opti-MEM™ (0.1% FBS). Data were analysed by 4 PL curve fitting in GraphPad® Prism 8. Three independent biological experiments were performed for each condition (Khandelia et al., 2024).

### 2.15 Confocal imaging of hOAAC treated with rhodamine-123 entrapped SLNs

hOAAC (6 × 10^4^/well) were seeded on coverslips in 6-well plates and cultured overnight at 37 °C in a humidified 5% CO_2_ incubator. Cells were washed with PBS and incubated for 3 h at 37 °C with Rhodamine-123–loaded SLNs (1.4 μg/mL R123 in Opti-MEM™). Following incubation, cells were washing twice with PBS and fixed in 4% formaldehyde for15 min at room temperature. Then the cells were washed with PBS and stained with Alexa Fluor® 594 (1 μg/mL) in HBSS for 10 min at 37 °C, followed by DAPI (300 nM) staining for30s at room temperature. Coverslips were washed in PBS, mounted usingMowiol (mounting medium), and allowed to cure for 1 h at room temperature prior to confocal imaging

## 3 Results

### 3.1 Synthesis of SLNs using hot homogenisation

Six SLNs NP formulations were synthesised using HH with a selection of parameters (e.g., lipid concentration, surfactant concentration, and drug-to-lipid ratio) (Tables 1, 2). Their characterisation is given in Table 3. The major factors influencing particle size, particle dispersion index (PDI), and zeta potential were the type and concentration of lipids and surfactants, as well as the drug-to-lipid ratio. JS Mulla et al., 2009 reported that increasing lipid concentration increases particle size and broadens the PDI (JS MULLA, 2009). Formulation 1 (F1) comprised 2.5% w/v tristearin, 0.5% w/v DOTAP, 0.5% w/v Tween 80, and a 0.2:1 drug-to-lipid ratio, which served as the highest-lipid baseline for comparison. F1 had a larger particle size and higher PDI of 366 nm and 0.35 than F2 of 327 nm and 0.25, suggesting that a 0.5% w/v increasing the lipid concentration from 2.0% to 2.5% w/v resulted in larger and more heterogeneous particles. F1–F4 exhibited higher particle sizes comparable to blank SLNs less than 250 nm, whereas F5 showed a higher particle size of 390 nm due to an increased drug-to-lipid ratio, since the drug was incorporated into the lipid matrix during SLN formation (Melike Üner, 2022). F2 and F3 exhibited the lowest PDIs (0.25 and 0.23, respectively), indicating a more uniform particle size distribution compared with F1, F4, and F5. F2 and F3 displayed PDIs similar to blank SLNs, whereas F1, F4, and F5 exhibited higher PDI values. The increased PDI observed for F4 and F5 may be associated with the higher drug-to-lipid ratios usd in these formulations, suggesting that increased drug loading could affect particle homogeneity and lipid matrix organisation. All formulations exhibited positive zeta potentials due to the cationic lipid, DOTAP, which should facilitate HA coating through electrostatic interaction. However, formulations F4 and F5 showed reduced zeta potentials compared with the other formulations from average 40 mV to 8.6 (F4) and 27 mV (F5), suggesting destabilisation at higher lipid to drug ratio. Based on these findings, F1 (final row in both tables) was selected for the synthesis of DXP-SLNs initially by HH (manual) and then for HA-DXP-SLNs using microfluidics. F1 demonstrated optimal physicochemical properties, including a uniform particle size distribution, low polydispersity index, and high positive zeta potential indicative of a stable and well-structured lipid matrix suitable for further optimisation.

**TABLE 1:** Composition rationale for materials used in the development of HA-DXP-SLNs.

Components in SLN formulation	Rationale
Tristearin	Tristearin was selected as the solid lipid matrix due to its physicochemical stability and ordered triglyceride structure, which supports formation of stable SLNs and enables incorporation of lipophilic drugs such as DXP for sustained release (Hughes and Walsh, 2015).
DOTAP	DOTAP is one of the most widely used cationic lipids. It generates a positively charged to SLNs surface for subsequent HA coating through electrostatic interaction (Godbey, 2014).
Tween® 80	Surfactant for SLNs formulation.
DMG-PEG 2000	Unlike Tween 80, which mainly functions as a surfactant during nanoparticle formation, DMG-PEG also provides a PEG surface layer that contributes to steric stabilisation. PEGylation has been reported to improve nanoparticle stability, reduce aggregation, and enhance particle retention by limiting surface interactions and opsonisation (Kakkar et al., 2015).
DXP	DXP was selected as the glucocorticoid component due to its high lipophilicity, which facilitates incorporation within the lipid matrix, while enzymatic conversion to active dexamethasone within the joint environment may enable sustained local drug release and prolonged anti-inflammatory activity following IA administration (Mountziaris et al., 2009).
HA	HA was incorporated as a surface coating material due to its biocompatibility, lubricating and anti-inflammatory properties, and its ability to interact with CD44-expressing inflammatory cells and chondrocytes within the OA microenvironment, while also improving nanoparticle surface stability and functionality (Kang et al., 2021; Saniya Salathia, 2023).

**TABLE 2:** Composition of six SLNs formulations using manual HH.

Formulation	Tristearin (% w/v)	DOTAP (% w/v)	Tween 80 (% w/v)	Drug: Lipid
Control-SLNs	2.5	0.5	0.5	N/A
F1 (DEX-SLNs)	2.5	0.5	0.5	0.2:1
F2 (DEX-SLNs)	2.0	0.5	0.5	0.2:1
F3 (DEX-SLNs)	2.0	0.5	0.5	0.4:1
F4 (DEX-SLNs)	2.0	0.5	0.5	0.5:1
F5 (DEX-SLNs)	2.0	0.5	0.5	0.6:1
F1 (DXP-SLNs)	2.5	0.5	0.5	0.2:1

**TABLE 3:** Characterisation of six SLNs formulations using manual HH.

Formulation	Size (nm)	PDI	Zeta (mV)	EE %	DL %
Control-SLNs	240 ± 11	0.25 ± 0.08	40 ± 7	N/A	N/A
F1 (DEX-SLNs)	366 ± 11	0.35 ± 0.06	43 ± 6	8.2 ± 1.2	0.8 ± 0.2
F2 (DEX-SLNs)	327 ± 18	0.25 ± 0.07	44 ± 8	1.4 ± 0.2	0.3 ± 0.02
F3 (DEX-SLNs)	301 ± 22	0.23 ± 0.01	48 ± 4	8.6 ± 0.2	3.4 ± 0.2
F4 (DEX-SLNs)	315 ± 19	0.33 ± 0.02	8.6 ± 2	1.5 ± 0.3	0.8 ± 0.3
F5 (DEX-SLNs)	390 ± 28	0.32 ± 0.02	27 ± 3	0.5 ± 0.02	0.3 ± 0.02
F1 (DXP-SLNs)	269 ± 13	0.32 ± 0.05	42 ± 5	4.1 ± 0.2	0.4 ± 0.2

### 3.2 Synthesis and characterisations of control and drug-loaded SLNs using microfluidics

**FIGURE 2:**
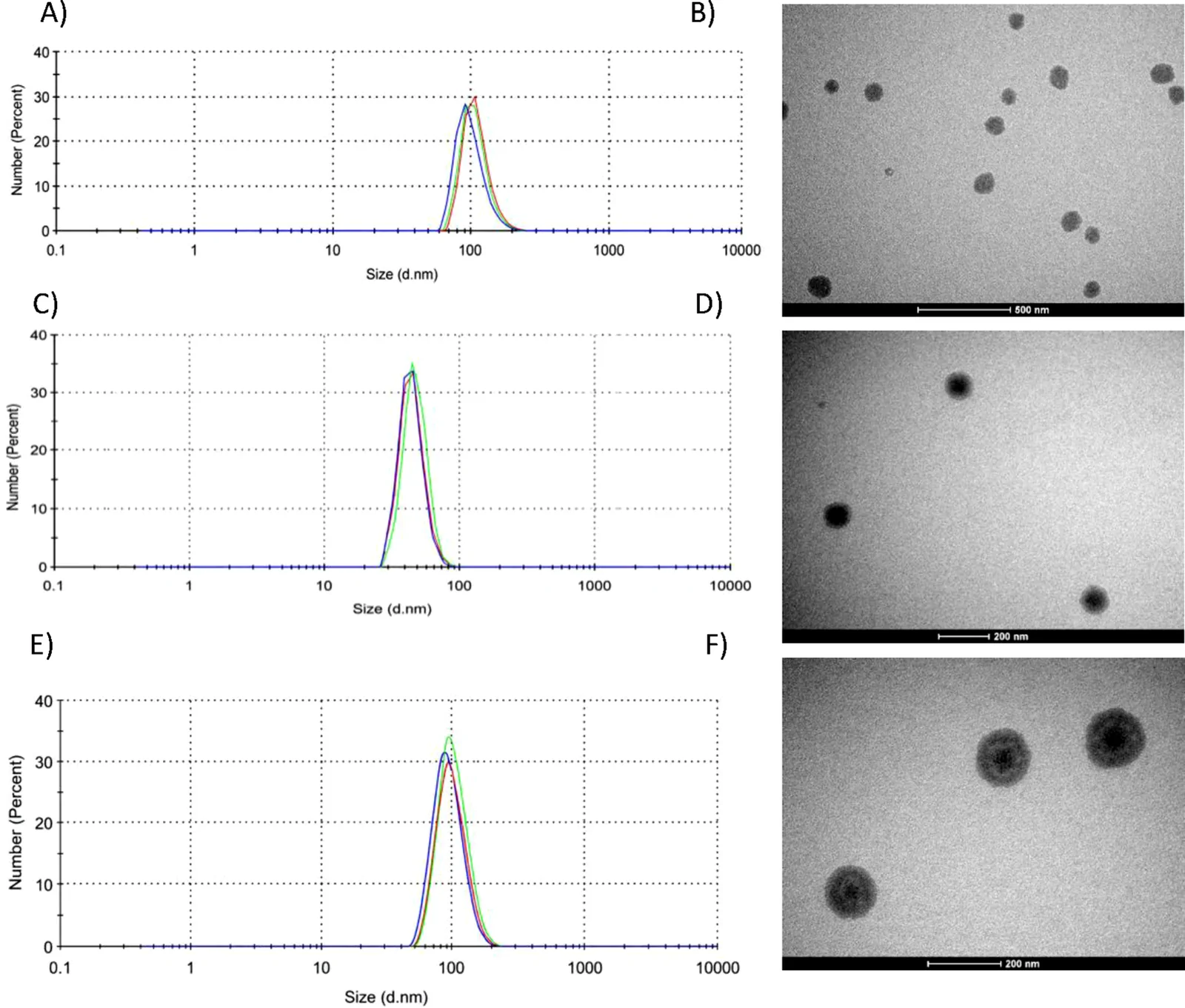
Particle size distribution by number and TEM images of control-SLNs **(A,B)**, DXP-SLNs **(C,D)**, and HA-DXP-SLNs **(E,F)** made by microfluidics at 25 °C.

**FIGURE 3:**
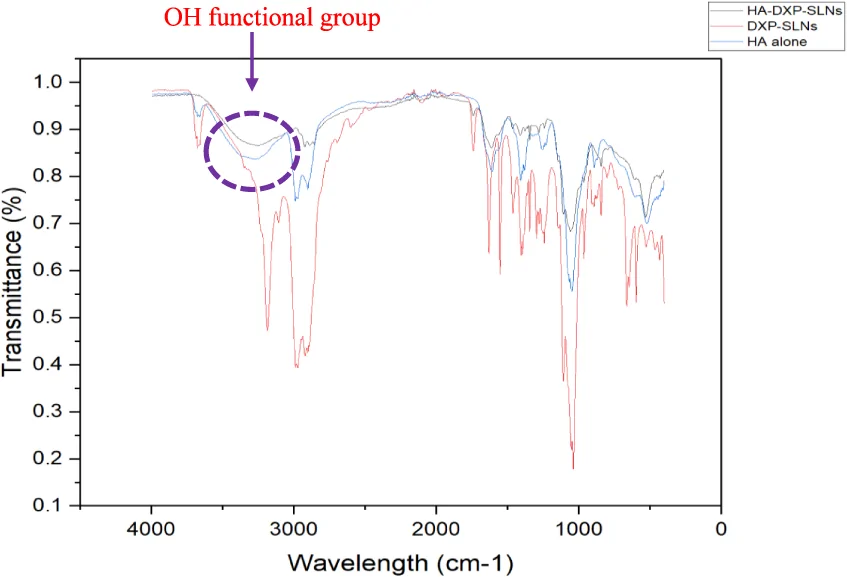
Attenuated Total Reflectance-Fourier Transform Infrared spectroscopy (ATR-IR) spectra for HA-DXP-SLNs. HA-DXP-SLNs (black), DXP-SLNs (red), and HA alone (blue).

DXP-SLNs prepared by microfluidics demonstrated greater reproducibility, higher encapsulation efficiency up to 84.3% for DEX-SLNs and 88.5% for DXP-SLNs, and narrower size 102 nm for DEX-SLNs and 57 nm for DXP-SLNs distribution than HH formulations (Figure 2; Tables 4, 5). HA coating of DXP-SLNs increased particle size to 90 nm, consistent with deposition of an additional HA layer, while the PDI decreased to 0.17, suggesting improved structural uniformity. Zeta potentials also shifted from positive 30.8 mV to negative −17.3 mV after HA coating, reflecting the presence of negatively charged carboxyl groups in HA. ATR-F spectra confirmed successful coating (Figure 3, and ELISA quantification indicated ∼0.45 mg HA per g of DXP-SLNs. *

**TABLE 4:** Composition of HA-DXP-SLNs using F1 parameters made with microfluidics.

Composition(s)	Tristearin	1,2-Dioleoyl-3-trimethylammonium propane (DOTAP)	DMG-PEG2000	HA
Concentration(s)	20 mg/mL	20 mg/mL	10 mg/mL	3% w/v
Condition	FRR	TFR		
	3(organic):1(aqueous)	15 mL/min		

TFR: total flow rate; FRR: flow rate ratio.

**TABLE 5:** Characterisation of control- and drug-loaded SLNs made by microfluidics.

Parameter	Control-SLNs	DEX-SLNs	DXP-SLNs	HA-DXP-SLN
Size (nm)	115 ± 10	102 ± 6	57 ± 6	90 ± 6
PDI	0.20 ± 0.04	0.21 ± 0.02	0.24 ± 0.03	0.17 ± 0.03
Zeta potential (mV)	+33 ± 2	+29 ± 3	+30.8 ± 5	−17.3 ± 2
Encapsulation efficiency (%)	N/A	84.3 ± 3	88.5 ± 2	89.7 ± 1
Drug loading (%)	N/A	5.6 ± 3	6.0 ± 1	6.3 ± 1
[HA] coated (mg/g)	N/A	N/A	N/A	0.42 ± 0.1*

**FIGURE 4:**
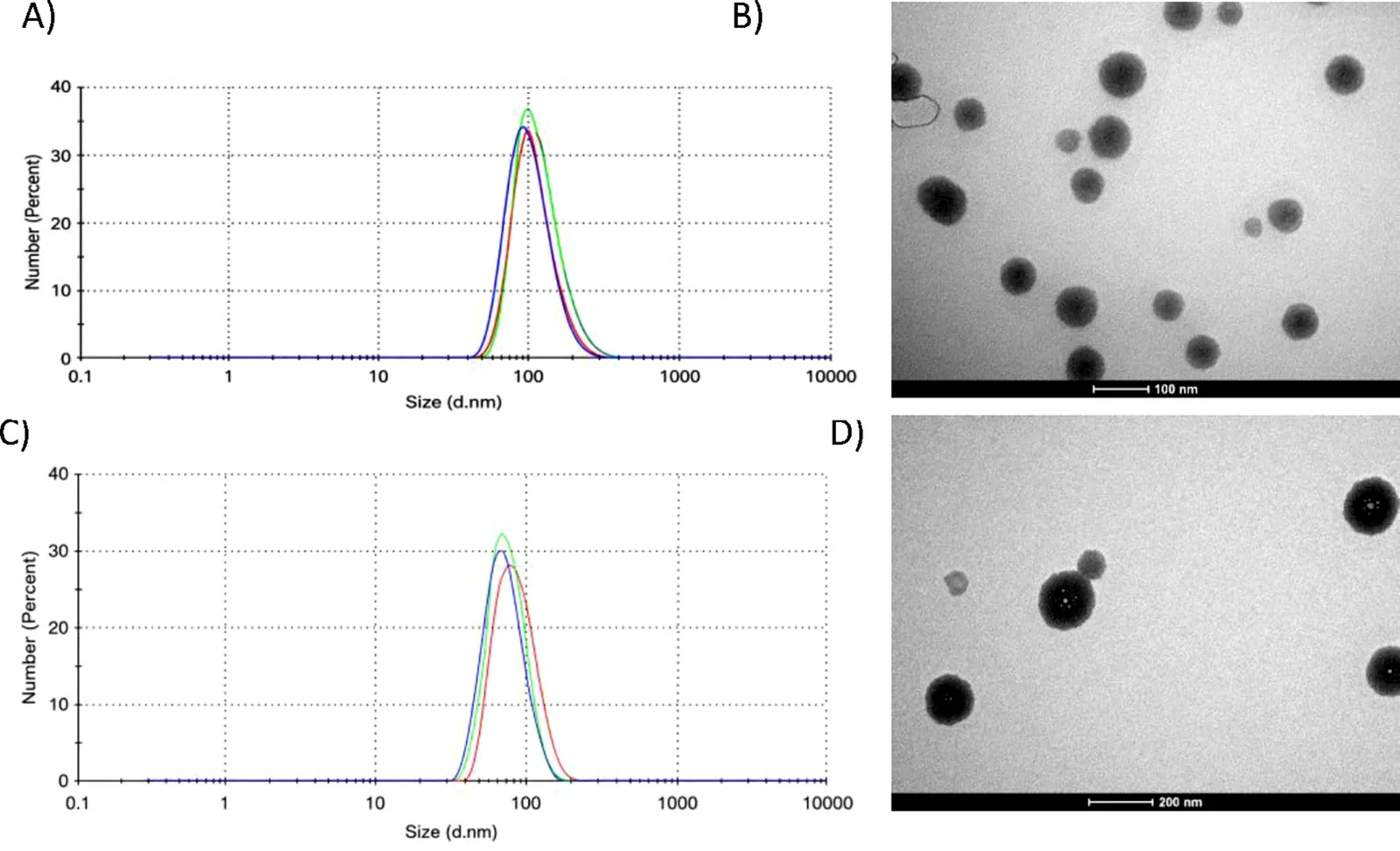
Stability of control-SLNs vs. HA-DXP-SLNs made by microfluidics over 4 weeks at 4 °C. Particle size distribution by number and TEM images of control-SLNs **(A,B)** and HA-DXP-SLNs **(C,D)**.

Other studies indicate that coating NPs with natural hydrophilic polymers, including HA can enhance the stability of NPs in aqueous solutions (Lee et al., 2019). Here, the average particle size and PDI of control-SLNs and HA-DXP-SLNs were assessed by DLS to evaluate the effect of HA coating on SLN stability (Figure 4). After 1 month incubation at 37 °C, the mean particle size of control-SLNs increased from 115 nm to 147 nm, with the PDI rising from 0.20 to 0.25. In contrast, HA-DXP-SLNs made by microfluidics maintained a stable particle size of ∼95 nm and PDI (0.19) over the same period. Encapsulation efficiency and loading of DXP were also enhanced by microfluidic synthesis and HA coating. HA-DXP-SLNs achieved an EE% of 89.7% and DL% of 6.3% w/w for DXP, outperforming the commercial liposomal DXP formulation Lipotalon® (DL = 4% w/w) (Mountziaris et al., 2009). Similar findings were reported by Zewail et al., Lee et al., and Meylina et al., who observed that HA coating enhances drug retention by stabilising the nanoparticle interface through electrostatic and hydrogen-bonding interactions. The carboxyl groups of HA interact with cationic components of the lipid matrix, forming a compact and cohesive surface layer that restricts drug diffusion from the core. This interfacial reinforcement reduces drug loss during processing and storage, resulting in a modest increase in encapsulation efficiency and drug loading. The hydrophilic HA shell also contributes to steric stabilisation and improved dispersion in aqueous media, further preserving particle integrity and drug entrapment (Zewail et al., 2022; Lee et al., 2019; Meylina et al., 2023).

### 3.3 Attenuated total reflectance-fourier transform infrared spectroscopy (ATR-IR) analysis for HA-DXP-SLNs

HA coating on DXP-SLNs made by microfluidics was inferred by characteristic peaks in the ATR-IR spectra (Figure 3). A symmetric carboxylate peak was observed at 1409 cm^−1^, and an asymmetric carboxylate peak appeared at 1610 cm^−1^ in both the HA and HA-DXP-SLNs spectra. A broad peak at 3,200 cm^−1^ corresponding to OH stretching was present in both HA alone and HA-DXP-SLNs spectra. Additionally, an ester C=O stretch peak at 1739 cm^−1^ was observed in both the DXP-SLNs and HA-DXP-SLNs spectra, which was attributed to the ester group in tristearin.

### 3.4 *In vitro* release of DXP from DXP-SLNs and HA-DXP-SLNs

**FIGURE 5:**
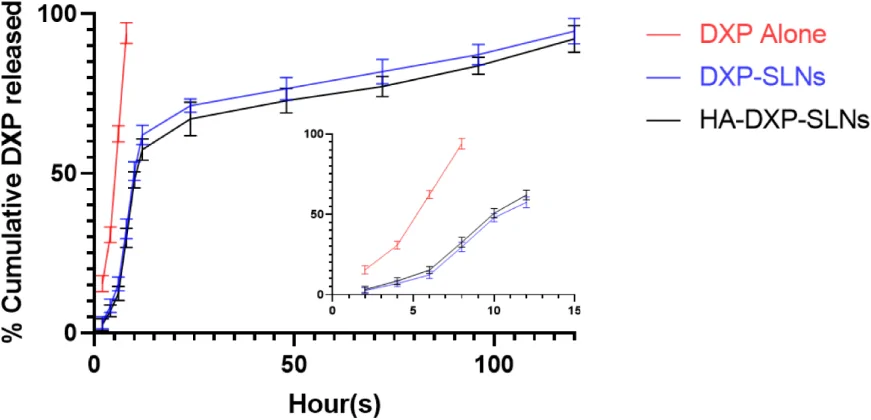
Cumulative release profile of DXP from DXP-SLNs and HA-DXP-SLNs over 120 h at 37 °C in PBS.

DXP release from solid lipid nanoparticles prepared by microfluidics, with or without surfacecoated HA, was quantified in PBS (pH 7.4) at 37 °C over 120 h using equilibrium dialysis (Figure 5). Both DXP-SLNs and HA-DXP-SLNs exhibited biphasic release kinetics, characterised by a burst phase within the first 8 h, followed by a diffusion dominated sustained release profile.

Free DXP rapidly diffused across the dialysis membrane, reaching 94.6% ± 2.3% cumulative release by 8 h. In contract, both nanoparticle formulations exhibited a slower release profile consistent with incorporation of DXP within the lipid matrix. Although the release profiles were broadly similar during the initial phase, HA-DXP-SLNs HA-DXP-SLNs generally exhibited lower cumulative release than DXP-SLNs throughout the study period. At 24 h, cumulative release reached ∼72–78% for DXP-SLNs and ∼65–71% for HA-DXP-SLNs, followed by continued gradual release. By 72 h, release approached ∼85–90% for DXP-SLNs and ∼78–84% for HA-DXP-SLNs. At the final 120 h timepoint, both formulations neared maximal release, reaching 95.9% ± 1.8% for DXP-SLNs and 93.5% ± 1.9% for HA-DXP-SLNs. This finding suggest that HA coating moderated the rate of DXP release without substantially affecting the overall extent of drug release.

### 3.5 *In vitro* measurement of DEX from DXP-NPs vs. HA-DXP-SLNs in mouse plasma

**FIGURE 6:**
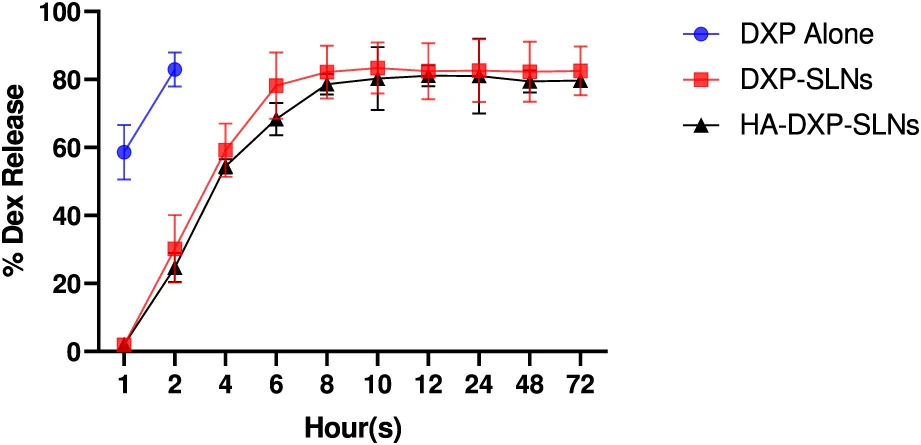
Cumulative release and conversion profile of DEX from DXP-SLNs and HA-DXP-SLNs over 72 h at 37 °C in mouse plasma.

*In vitro* conversion of DEX from DXP-SLNs and HA-DXP-SLNs made by microfluidics were evaluated in murine plasma at 37 °C over 72 h (Figure 6). Both formulations exhibited a biphasic release pattern characterized by a gradual release phase over 6 h, followed by a plateau. Free DXP (DXP alone) showed a rapid enzymatic conversion to DEX, reaching approximately 80% release within 2 h. After 1 h, approximately 22.3% ± 3.1% of DXP was released from DXP–SLNs compared with 18.7% ± 2.8% from HA–DXP–SLNs. By 6 h, cumulative release reached 74.5% ± 4.2% and 66.8% ± 5.1% for DXP–SLNs and HA–DXP–SLNs, respectively. Thereafter, release rates gradually levelled off. At the 72-h time point, the cumulative values of DEX were 81% (DXP–SLNs) and 79% (HA–DXP–SLNs), with no significant difference observed between the two groups (p > 0.05).

### 3.6 Effects of SLNs made by microfluidics on viability of THP-1 cells: MTS assay

**FIGURE 7:**
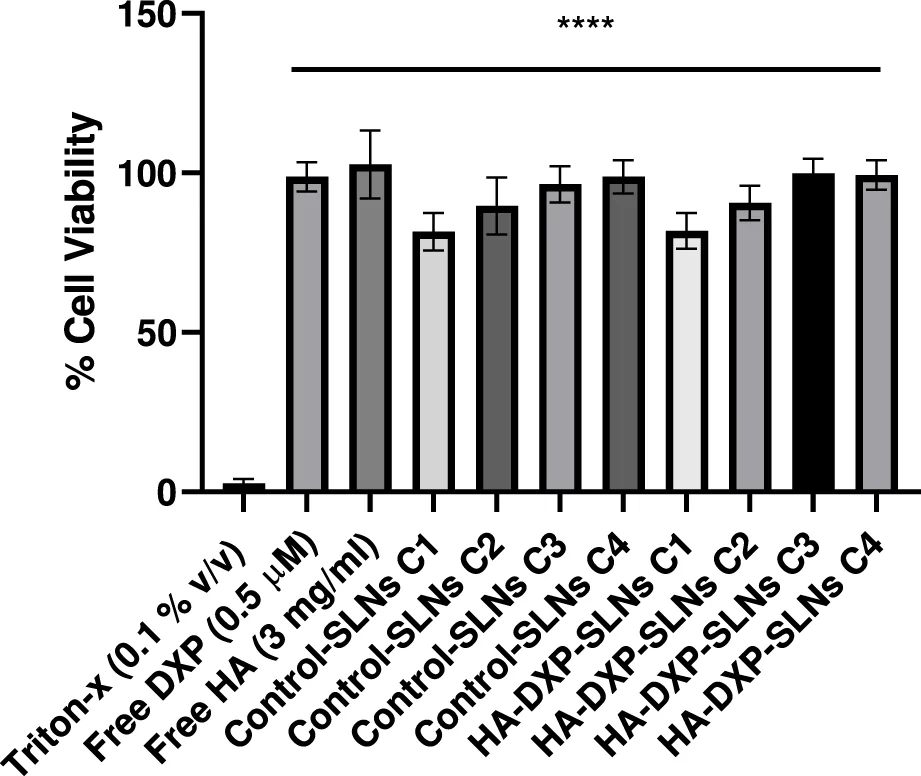
Effect of HA-DXP-SLNs vs. free DXP and HA on THP-1 cells on mitochondrial function as measured by MTS assay following exposure for 24 h. The concentration of control-SLNs was equivalent to the concentration of HA-DXP-SLNs in 0.125–0.75 µM C1 is 0.75 μM, C2 is 0.50 μM, C3 is 0.25 μM, C4 is 0.125 μM. Triton®-X-100 (0.1% v/v) was the positive control. Data expressed as mean ± SD, n = 3 per group. For statistical analysis, a one-way ANOVA with Dunnett’s *post hoc* test was used. *p < 0.05; **p < 0.01; ****p < 0.001 compared to Triton-X-100.

To assess mitochondrial function in THP-1 cells, an MTS assay was performed. As shown in Figure 7, SLNs demonstrated good biocompatibility, with THP-1 cell viability decreasing only slightly from 99% to 81% as the concentration of control and HA-DXP-SLNs increased from 0.125 µM to 0.75 µM. The concentration of control-SLNs correspond to the molar amount of encapsulated DXP, based on measured drug loading. Drug free control-SLNs were applied at the equivalent nanoparticle mass/volume associated with each HA-DXP-SLN dose, allowing direct comparison of nanoparticle related effects. Neither free DXP (0.5 µM) nor HA (3 mg/mL) induced cytotoxicity after 24 h of incubation.

### 3.7 Effects of SLNs made by microfluidics on PGE2 secreted by LPS-stimulated THP-1 cells

**FIGURE 8:**
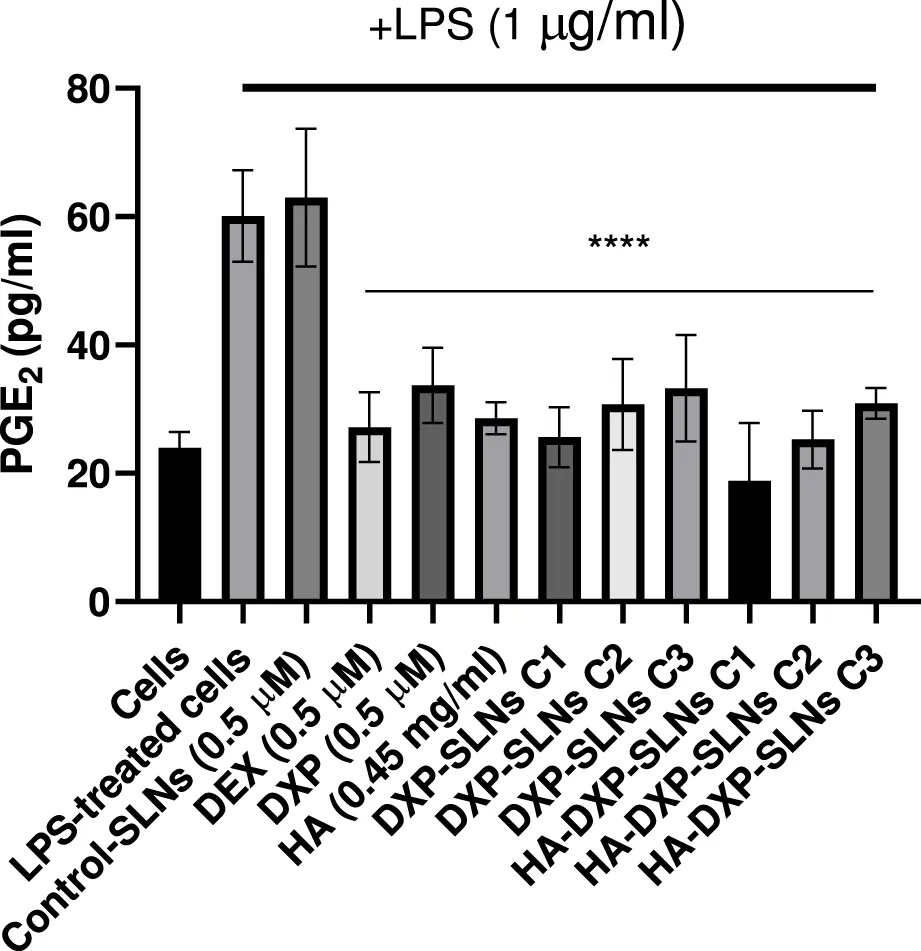
Reduction in PGE_2_ concentrations released by LPS-stimulated THP-1 cells by HA-DXP-SLNs. The concentration of control-SLNs was equivalent to the concentration of HA-DXP-SLNs in 0.5 μM C1 is 0.5 μM, C2 is 0.25 μM, and C3 is 0.125 μM. Bars represent the mean ± SD of three biological experiments; the three data points per group represent the mean of each biological experiment. For each biological experiment, three technical replicates were obtained. For statistical analysis, a one-way ANOVA with Dunnett’s *post hoc* test was used. *p < 0.05; **p < 0.01; ****p < 0.001 compared to LPS-stimulated only (second column from the left). Cells: THP-1 cells cultured just in media. LPS-stimulated cells: without nanoparticles or drug treatments (controls).

PGE_2_ concentrations were measured in media from LPS-stimulated THP-1 cells treated with or without HA-coated and DXP-loaded SLNs (Figure 8). Stimulating THP-1 cells with 1 μg/mL LPS increased the PGE_2_ concentration secreted into culture media (positive control). LPS-stimulated release of PGE_2_ was significantly decreased by all three concentrations of DXP in DXP-SLNs and in HA-DXP-SLNs. These reductions were observed when THP-1 cells were pretreated with both DXP-SLNs and HA-DXP-SLNs for 120 min before being stimulated with 1 μg/mL LPS. Importantly, HA-DXP-SLNs at all three concentrations reduced PGE_2_ levels more effectively than DXP-SLNs, demonstrating the potential benefit of the HA coating. In conclusion, HA-DXP-SLNs reduced PGE_2_ secretion in LPS-stimulated THP-1 cells, suggesting modulation of the COX/PGE_2_ pathway associated with inflammatory signalling. PGE_2_ is recognised as an important mediator in OA pathophysiology and has been associated with pain signalling, cartilage catabolism, subchondral bone remodelling, and inflammatory responses within the joint environment (Yang et al., 2024). The observed reduction in PGE_2_ secretion following exposure to HA-DXP-SLNs suggests that incorporation of DXP within the SLN system did not compromise glucocorticoid associated biological activity and may reflect release of DXP from the lipid matrix followed by enzymatic conversion to active DEX within the inflammatory environment. However, modulation of PGE_2_ secretion alone does not demonstrate a broad anti-inflammatory effect, and additional inflammatory and OA-associated biomarkers would be required to further confirm wider anti-inflammatory activity.

### 3.8 Effects of HA-DXP-SLNs on the cellular viability of hOAAC: MTS assay

**FIGURE 9:**
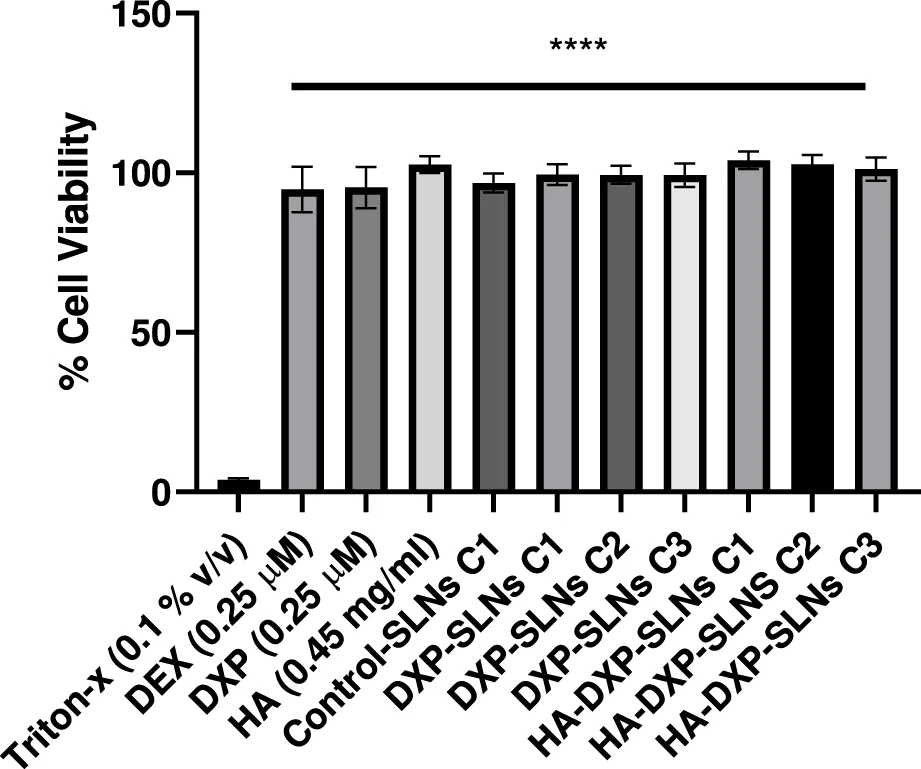
Effect of control-, DXP-SLNs, and HA-DXP-SLNs vs. free DXP and HA on mitochondrial function of hOAAC as measured by MTS assay following exposure for 24 h. Triton®-X-100 (0.1% v/v) was the positive control. C1 is 0.5 μM, C2 is 0.25 μM, and C3 is 0.125 μM. Data expressed as mean ± SD, n = 3 per group. For statistical analysis, a one-way ANOVA with Dunnett’s *post hoc* test was used. *p < 0.05; **p < 0.01; ****p < 0.001 compared to Triton-X-100 (0.1% v/v).

To evaluate the biocompatibility of the formulations, the mitochondrial activity of hOAAC was assessed using the MTS assay. As shown in Figure 9, exposure to DXP (0.25 µM), HA (0.45 mg/mL), control SLNs, DXP-SLNs, and HA-DXP-SLNs at three different concentrations (C1–C3) for 24 h resulted in cell viabilities above 95%, indicating excellent cytocompatibility. In contrast, the positive control (Triton X, 0.1% v/v) reduced cell viability to approximately 3%. These results confirm that none of the free DEX, DXP, HA, nor the nanoparticle formulations exerted cytotoxic effects on THP-1 cells under the tested conditions.

### 3.9 Effects of HA-DXP-SLNs on PGE_2_ levels of LPS-stimulated hOAAC

**FIGURE 10:**
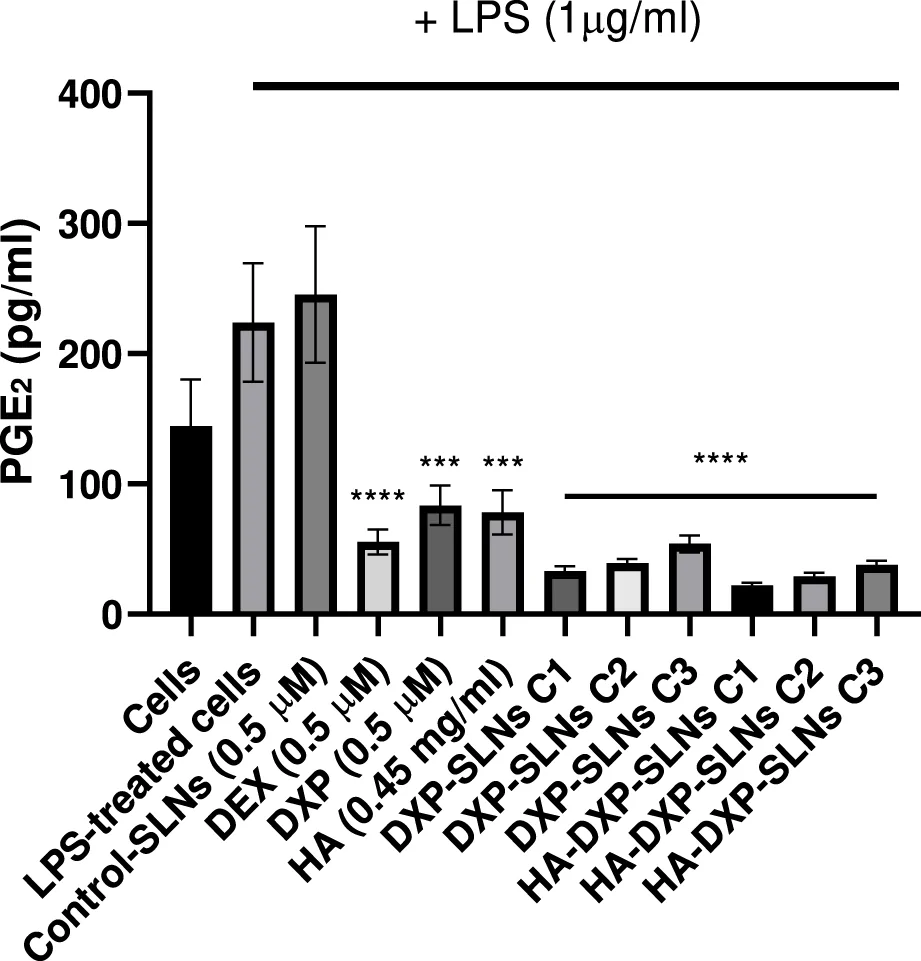
Reduction in PGE_2_ concentrations released by LPS-stimulated hOAAC by HA-DXP-SLNs. The concentration of control-SLNs was equivalent to the concentration of HA-DXP-SLNs in 0.5 μM C1 is 0.5 μM, C2 is 0.25 μM, and C3 is 0.125 μM. Bars represent the mean ± SD of three biological experiments; the three data points per group represent the mean of each biological experiment. For each biological experiment, three technical replicates were obtained. For statistical analysis, a one-way ANOVA with Dunnett’s *post hoc* test was used. *p < 0.05; **p < 0.01; ****p < 0.001 compared to LPS-stimulated only (second column from the left). Cells: hOAAC cells cultured just in media. LPS-stimulated cells: without nanoparticles or drug treatments (controls).

Stimulating hOAAC with 1 μg/mL LPS increased PGE_2_ concentration by 33% over basal (Figure 9). The released PGE_2_ concentrations were significantly decreased by all three concentrations of DXP in DXP-SLNs and HA-DXP-SLNs compared to cells stimulated with LPS alone. This reduction was observed when hOAAC were pretreated with either DXP-SLNs or HA-DXP-SLNs for 120 min before being stimulated. Additionally, HA-DXP-SLNs at all three DXP concentrations reduced PGE_2_ levels more effectively than DXP-SLNs (Figure 10). PGE_2_ has been implicated in OA associated inflammatory signalling, cartilage degradation, and pain related pathways (Yang et al., 2024). Therefore, the observed reduction in PGE_2_ secretion following treatment with HA-DXP-SLNs suggests modulation of the COX/PGE_2_ pathway in hOAAC. This effect may be associated with release of DXP from the SLN system and subsequent enzymatic conversion to active DEX within the inflammatory environment. The comparable biological response observed in both hOAAC and THP-1 cells further suggests that incorporation of DXP into the SLN formulation did not compromise glucocorticoid-associated biological activity.

### 3.10 Confocal imaging of hOAAC treated with R_123_-rhodamine-123 loaded SLNs

**FIGURE 11:**
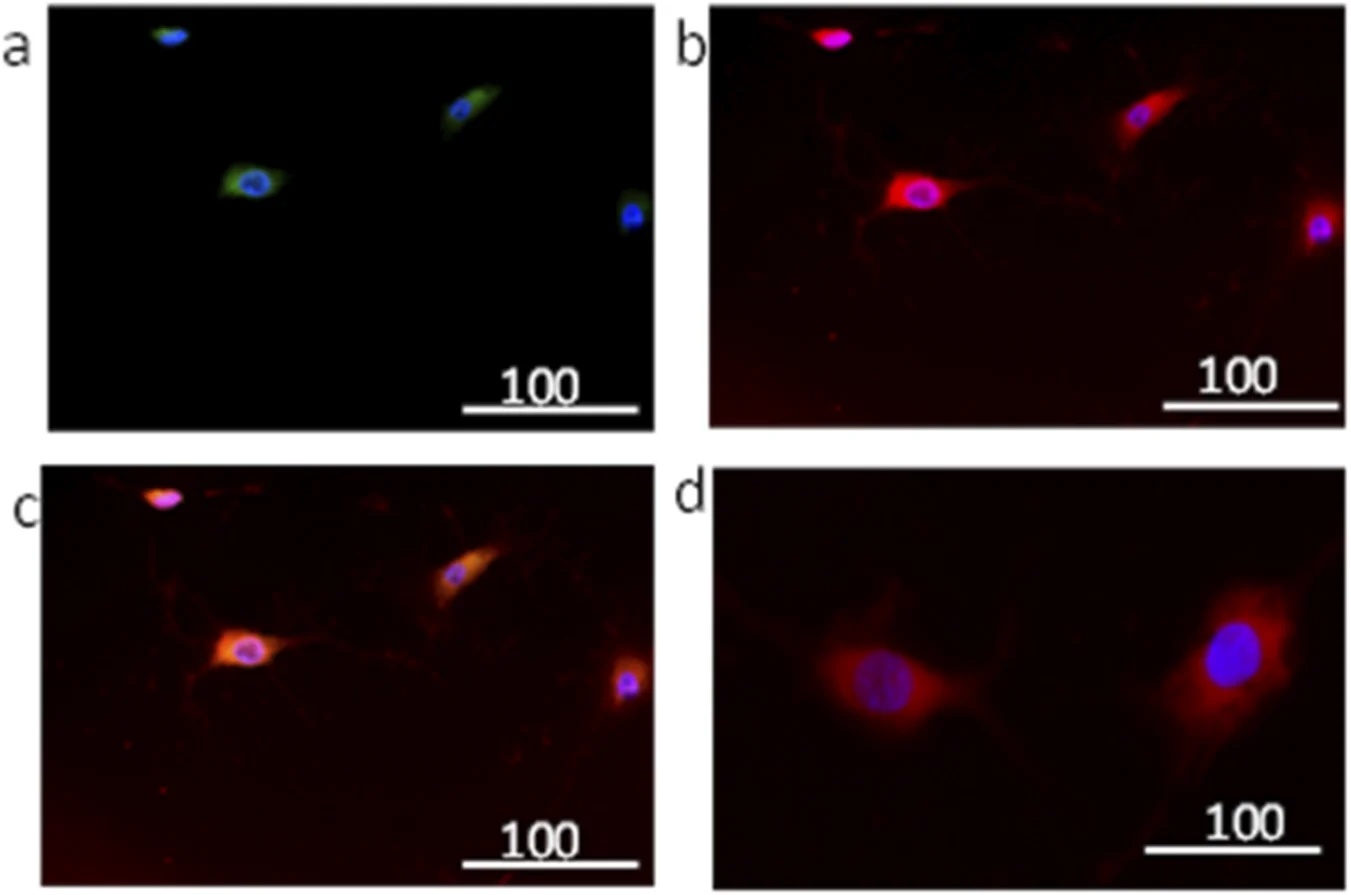
Confocal images of hOAAC after 4 h exposure to **(a)** Rhodamine-123-loaded SLNs (green) with nuclei **(b)** cytoskeleton labelled by Alexa Fluor®594 (red) and nuclei by DAPI (blue) **(c)** R123-loaded SLNs (green) with cytoskeleton (red) and nuclei (blue); **(d)** untreated control with cytoskeleton (red) and nuclei (blue).

To investigate the cellular uptake of SLNs, hOAAC were incubated with rhodamine-123-loaded SLNs and visualised by confocal microscopy. As shown in Figure 11D, untreated control cells exhibited cytoskeletal and nuclear staining, confirming the absence of autofluorescence. In contrast, cells treated with rhodamine-123-loaded SLNs displayed green fluorescence, indicating internalisation of nanoparticles. The overlay images revealed intracellular localisation of SLNs surrounding the nuclei, with fluorescence signals distributed throughout the cytoplasm. Figure 11 (c) demonstrated successful separation of nuclear, cytoskeletal, and nanoparticle signals, confirming that the observed intracellular green fluorescence corresponded to internalised rhodamine-123-loaded SLNs. These results demonstrate uptake of SLNs by hOAAC within 4 h of exposure.

## 4 Discussion

The present study demonstrates that HA-DXP-SLNs prepared by microfluidics represent a stable and biologically active formulation with anti-inflammatory potential. These findings are consistent with previous work showing that surface modification of lipid nanoparticles with naturally derived polymers, such as HA, can improve colloidal stability and facilitate receptor-mediated uptake by chondrocytes and macrophages (Zhou et al., 2018; Yu et al., 2019; Zhong et al., 2021). In addition to this targeting function, HA appears to contribute to drug retention and local bioactivity, as reflected by the greater suppression of PGE_2_ release observed here compared with uncoated particles. Incorporation of the lipophilic prodrug, DXP with the HA coating, may eventually support a sustained-release profile when optimised, addressing a major limitation of current intra-articular therapies (Zhou et al., 2018; Lorscheider et al., 2019; Ayhan et al., 2014).

To understand how these functional advantages arise from formulation design, it is important to consider the physicochemical principles governing nanoparticle formation. The formation of SLNs is governed by interfacial and surface tension between organic and aqueous phases. Adhesive forces between the phases are typically weak, resulting in higher surface tension than interfacial tension. During agitation, the system tends to minimise surface free energy by forming spherical particles, while surfactants lower the interfacial tension between the phases and help maintain nanoparticle stability. The extent of drug incorporation into the lipid matrix is influenced by both the formulation composition and the conditions under which the nanoparticles are formed. As a result, variations in EE% and DL% were observed across the HH formulations (Table 3). F1 displayed higher EE% and DL% of 8.2% and 0.8% than F2 of 1.4% and 0.3%, likely due to increased lipid concentration providing a larger matrix for drug incorporation (Muller et al., 2022). Increasing the drug-to-lipid ratio enhanced the EE% and DL% up to a threshold, beyond which stability was compromised. Lipid type also influenced EE% and DL% because of differences in crystal packing. Previous studies have shown that the composition and crystallinity of the lipid matrix can affect both nanoparticle stability and drug encapsulation. Lipid matrices with lower crystallinity may allow greater incorporation of lipophilic compounds, whereas more ordered crystalline structures can limit drug incorporation within the particle core (Shah, 2018; Duan et al., 2020).

The NanoAssemblr™ platform further improved SLN synthesis focussing on the FI prototype that emerged from manual synthesis by HH. Repeated splitting and merging of fluid streams enabled rapid, uniform mixing of organic and aqueous phases, resulting in NPs with smaller sizes, narrower distributions, higher reproducibility, and superior encapsulation compared with HH-derived SLNs (Zhang et al., 2023; Ali et al., 2021). TFR and FRR are key microfluidic parameters that influence nanoparticle characteristics. Roces et al., 2020 examined the influence of FRR (organic: aqueous phase from 1:1 to 5:1) and the TFR (5–20 mL/min) on lipid nanoparticles using the Ignite™ system.

Their study showed that increasing both FRR and TFR led to reduction in particle size up to a certain point, after which further increases produced little additional change in particle diameter (Roces et al., 2020). In this study, DMG-PEG 2000 was selected as the surfactant for Ignite™ formulations. PEGylation of the SLN surface may improve colloidal stability through steric stabilisation provided by the PEG surface layer. Previous studies using PEGylated SLNs have also reported reduced particle aggregation and altered particle surface interactions compared with conventional surfactant based systems (Kakkar et al., 2015). Similar findings have also been reported for PEGylated SLNs produced using microfluidic techniques, where PEG-containing formulations demonstrated narrow size distributions and stable nanoparticle dispersions (Arduino et al., 2021). In the present study, DMG-PEG 2000 was incorporated to support nanoparticle stability and facilitate incorporation of the lipophilic DXP within the lipid matrix.

In our study, the particle size of DXP-SLNs increased after HA coating, consistent with the formation of a HA layer around the NP surface through electrostatic interaction. Of note, the PDI decreased upon coating, which may reflect a possibly more compact stabilised structure resulting from electrostatic interactions between the cationic DXP-SLN surface and the negatively charged HA (Lee et al., 2019). This observation aligns with previous reports which attributed the enlargement in particle size following HA modification to the hydrated HA chains extending into solution, thereby increasing the hydrodynamic diameter of the nanoparticles (Yu et al., 2019; Zhou et al., 2018). Similarly, Zhong et al., demonstrated a size increase from 160 nm to 216 nm after HA functionalisation, confirming the successful deposition of HA on the nanoparticle surface (Zhong et al., 2021).

The findings from ATR-FTIR analysis also support the successful coating of HA onto DXP-SLNs, as indicated by characteristic surface vibrational signatures. In the HA-DXP-SLN spectrum, a broad absorption band appeared across the 3,000–3,600 cm^−1^ region, corresponding to O–H stretching vibrations. This band was more intense than in the uncoated DXP-SLNs and closely matched the O–H stretching envelope of HA alone, indicating the presence of HA derived hydroxyl and amide functionalities at the nanoparticle surface. In addition, characteristic carbohydrate associated bands attributed to HA were observed in the HA-DXP-SLN spectrum, further supporting successful surface coating. The spectral differences between uncoated DXP-SLNs and HA-DXP-SLNs indicate surface modification following HA incorporation.

These results are consistent with previous studies describing HA functionalisation nanoparticles systems, including HA-coated chitosan nanoparticles by Meylina et al. for anticancer applications in breast cancer (Meylina et al., 2023), HA-functionalised magnetic nanoparticles by Răcuciu et al. (Racuciu et al., 2024) and HA-coated nanostructured lipid carriers by Zewail et al. for the management of rheumatoid arthritis (Zewail et al., 2022). Our findings also closely parallel the ATR-FTIR results reported by Motamedifar et al. who demonstrated successful HA coating of gemcitabine-loaded magnetic SLNs. Their HA-coated nanoparticles showed strong O–H/N–H stretching bands within the 3,000–3,600 cm^−1^ region and characteristic carbohydrate-associated peaks at ∼1,609 cm^−1^ and ∼1,038 cm^−1^, which were markedly more intense compared to uncoated controls. The similarity between the HA-DXP-SLN spectra and those reported in Motamedifar et al. strengthens the conclusion that HA is successfully deposited as a surface layer in the present system (Motamedifar et al., 2025).

Drug release from SLNs is generally thought to occur through a combination of diffusion, lipid matrix erosion, and nanoparticle degradation, although the contribution of each mechanism depends on the properties of the formulation. Previous studies have reported that release of lipophilic drugs from SLNs is often mainly driven by diffusion through the lipid matrix, while factors including lipid crystallinity, surfactant composition, particle size, and matrix organisation can also influence release behaviour. Enzymatic degradation of lipid based nanoparticles under physiological conditions has also been suggested to contribute to drug release (Mishra et al., 2018; Manjunath et al., 2005). Therefore, the sustained release profile observed for HA-DXP-SLNs in the present study is likely due to a combination of these processes rather than a single release mechanism. The release profile of SLNs generally shows a burst release followed by a controlled release phase. Burst release is due to the initial release of weakly surface-bound drug, thereafter the drug slowly diffuses from the lipid matrix due to degradation or erosion of that matrix (Attama and Umeyor, 2015). The release behaviour captured in Figure 4 closely mirrors the diffusion retarded biphasic profiles described in similar SLN formulations. Where Chen et al. where reported that Q10-loaded SLNs released ∼50–60% of drug in the first 4–6 h, followed by slower diffusion through the lipid matrix, reaching ∼96% cumulative release by 24 h, which they interpret as early desorption from the nanoparticle interface prior to matrix-retarded efflux (Atapour-Mashhad et al., 2024). Patra et al. similarly observed that chlorambucil-loaded SLNs exhibited an initial burst phase of ∼50% within the first 2–4 h, followed by sustained release to >90% by ∼21 h at 37 °C in PBS, with kinetic fitting supporting diffusion as the dominant release mechanism through a solid glyceride core (Patra et al., 2024). Martínez et al. showed that steroid-loaded SLNs restricted premature membrane diffusion, limiting early release to ∼10% within 2–4 h in mouse plasma, before permitting sustained release, emphasising that low burst magnitude reflects lipid-core confinement of the prodrug prior to buffer or enzyme access Ayesha et al. reported that diacerein-loaded SLNs demonstrated 35%–55% release in the first 6 h, with 85% diffusing out by 24 h (Ayesha et al., 2025). Lee et al. similarly observed ∼40% docetaxel release from SLN within 4 h, followed by a gradual release over 72 h (Lee et al., 2019). In both studies, HA coatings reduced the later-phase release, consistent with HA providing an additional diffusional barrier at the particle surface. Zhou et al. confirmed this with prednisolone-entrapped SLNs, whereby HA-coated formulations released only ∼38% by 30 h *versus* ∼56% from uncoated particles(Zhou et al., 2018). Chemistry of the steroid is also important in the SLN design. Both Chen et al. and Bae et al. showed that converting DEX to DXP reduced premature leakage from SLN, likely because the hydrophobic prodrug anchors in the lipid matrix (49, 50). Moreover, free DEX leaked ∼90% within 24 h, while DXP-loaded SLNs released only ∼15% DEX over 48 h (Chen et al., 2024; Bae et al., 2024). Lorscheider et al. extended this observation with PEGylated DXP nanoparticles, which maintained stability, high loading, and enzymatic conversion to active DEX in plasma (Lorscheider et al., 2019). As in the current study, both Bae and Lorscheider used mouse plasma rather than a simple buffer for release testing. DXP requires enzymatic cleavage by plasma esterases to generate pharmacologically active DEX. Although simulated synovial fluid can approximate the ionic strength and viscosity of the joint microenvironment, it lacks the full complement of esterases and lipolytic enzymes present *in vivo* and therefore cannot accurately reproduce the metabolic activation of DXP to DEX, hence we did not use it here. As highlighted by Lorscheider et al., testing in plasma therefore provides a representative assessment of enzymatic conversion capacity prior to direct assessment in knee joints (Bae et al., 2024). Our study therefore used mouse plasma for profiling DXP-SLNs and HA–DXP–SLNs for the enzymatic conversion to DEX.

Taken together, these results underscore several points. First, SLNs consistently transform free drug’s rapid dissolution into a controlled release profile, which can be further tuned by surfactant concentration, temperature, and particle size. Second, surface coating with HA slows release and potentially adds targeting capacity, while prodrug strategies also control of premature leakage. Third, plasma-based assays are essential when evaluating ester prodrugs since they directly measure the biologically relevant generation of active drug. While the *in vitro* plasma assay indicated near-complete release of DXP within 72 h, this finding should not be interpreted as equivalent to rapid clearance from the joint *in vivo*. In the synovial environment, several factors are likely to prolong effective drug exposure beyond this window, including restricted nanoparticle diffusion through the viscous synovial matrix and limited lymphatic clearance of ∼90 nm particles (Ayhan et al., 2014; Sladek et al., 2018; Brown et al., 2019). The hydrophobic nature of DXP also favours partitioning within lipid-rich tissue compartments, supporting gradual enzymatic activation *in situ* (Lorscheider et al., 2019; Bae et al., 2024). Moreover, HA coating can further promote binding to cartilage and synovial components *via* CD44 and electrostatic interactions, creating a local depot that sustains anti-inflammatory activity (Craciunescu et al., 2021; Zhou et al., 2018; Yu et al., 2019). In the present study, HA coating improved nanoparticle stability and reduced the initial burst release of DXP *in vitro*. The72-h plasma release profile mainly reflects enzymatic activation and release behaviour under sink conditions. Further *in vivo* studies are needed to determine whether HA coating influences IA retention or pharmacodynamic activity following joint administration.

Monocytes and macrophages contribute to inflammatory signalling in OA through the production of cytokines, prostaglandins, and other inflammatory mediators (Guillem-Llobat et al., 2024). THP-1 monocytes were therefore used in this study as an initial *in vitro* inflammatory model to assess the effects of HA-DXP-SLNs on LPS-induced PGE_2_ secretion, prior to evaluation in primary hOAAC, which represent a more disease-relevant cell model for OA associated cartilage pathology. The present study demonstrates that SLNs exhibit excellent biocompatibility with THP-1 cell viability remaining above 80% at concentrations up to 0.75 µM. Importantly, neither high free DXP (0.5 µM) nor HA (3 mg/mL) concentration exerted cytotoxic effects. Although cationic lipids such as DOTAP are often associated with cytotoxicity at high concentrations, their incorporation at low levels within the SLNs matrix and subsequent shielding by PEG and HA layers likely mitigated membrane disruption and charge-mediated toxicity (Lee et al., 2019; Zhou et al., 2018; Yu et al., 2019). This is consistent with previous findings that PEGylation and HA functionalisation markedly reduce the inherent cytotoxicity of cationic lipid-based carriers. Moreover, the selected concentrations of control SLNs, DXP-SLNs, and HA-DXP-SLNs also demonstrated excellent biocompatibility in hOAAC derived from OA patients.

LPS, a key component of Gram-negative bacterial cell walls, is known for its capacity to activate monocytes and macrophages (Tucureanu et al., 2018). Glucocorticoids inhibit many proinflammatory response genes through the repression of proinflammatory transcription factors (such as activator protein-1 and nuclear factor), as well as through the induction of anti-inflammatory protein synthesis (Smoak and Cidlowski, 2004; Chang et al., 2021). Exposure to both DXP-SLNs and HA-DXP-SLNs led to a marked reduction in PGE_2_ release from both LPS-stimulated THP-1 cells and hOAAC, consistent with the capacity of glucocorticoids to suppress proinflammatory transcription factors and promote anti-inflammatory protein synthesis (Smoak and Cidlowski, 2004; Chang et al., 2021). The enhanced suppression observed following 120 min of SLNs exposure suggests that intracellular uptake and sustained drug release play a key role in the observed effect. HA-DXP-SLNs reduced PGE_2_ levels more effectively than DXP-SLNs in LPS-stimulated monocytes and hOAAC, indicating superior anti-inflammatory activity of the combination of DXP with HA. These findings align with prior work showing that HA functionalisation enhances therapeutic efficacy through CD44 receptor-mediated targeting. For instance, Zhou et al. reported that HA-coated SLNs carrying prednisolone achieved higher joint accumulation and significantly greater therapeutic efficacy than uncoated SLNs in a collagen-induced arthritis model (Zhou et al., 2018).

A similar trend has been reported in oncology models, where HA decorated Pluronic®-85- coated SLNs loaded with paclitaxel achieved the greatest inhibition of tumour growth compared with uncoated paclitaxel-loaded SLNs formulations (Wang et al., 2017). Similarly, Zewail et al. also reported that HA-coated teriflunomide lipid nanocarriers produced a four-fold reduction in TNF-α serum levels normalised cartilage structure and restored hyaline cartilage integrity in rodent rheumatoid arthritis models compared with suspension formulations (Zewail et al., 2022). Zhong et al. further demonstrated that HA-coated methotrexate-linked branched polyethyleneimine nanoparticles selectively targeted activated macrophages *via* CD44-mediated endocytosis and reduced TNF-α and IL-6 in the joints of collagen-induced arthritis models (Zhong et al., 2021). Similarly, Yu et al., developed HA-coated, acid-sensitive DEX-entrapped nanoparticles that showed selective uptake by activated macrophages, reductions in TNF-α, IL-6, and cartilage/bone destruction in an adjuvant-induced arthritic rat model (Yu et al., 2019).

Confocal microscopy confirmed intracellular uptake of rhodamine _123_-loaded SLNs by hOAAC, with fluorescence mainly distributed within the cytoplasm region. This indicates that the SLNs were able to interact with and be internalised by OA associated chondrocytes under the conditions used in this study. Similar intracellular localisation of fluorescently labelled SLNs has also been reported in other cell types, suggesting that SLNs can readily undergo cellular uptake lines (Martins et al., 2012; Lee et al., 2019). Importantly, the absence of significant background fluorescence in untreated controls rules out potential artefactual signals arising from free rhodamine _123_ or unloaded nanoparticles (Martins et al., 2012). Our findings are in agreement with Martins et al. who reported similar intracellular accumulation of rhodamine _123_-loaded SLNs in glioma and macrophage cells after short incubation periods. As in their study, the clear separation of nanoparticle, cytoskeletal, and nuclear signals in our controls rules out the possibility that free rhodamine_123_ contributed to fluorescence. The comparable uptake profile between hOAAC and other mammalian cells suggests that SLNs are capable of efficient cellular entry across different cell types (Martins et al., 2012).

## 5 Conclusion

This work establishes HA-DXP-SLNs as biocompatible structures with anti-inflammatory efficacy in OA relevant cells. By demonstrating efficient uptake and greater suppression of PGE_2_ release compared with uncoated SLNs in hOAAC, our findings highlight the active contribution of HA to efficacy, going beyond its conventional role as a stabiliser. This represents a novel extension of the use of HA functionalised nanocarriers, previously validated in oncology and rheumatoid arthritis, into the context of primary osteoarthritic chondrocytes, Compared with recent studies on glucocorticoid-loaded SLNs, our results underscore the combined advantages of prodrug stabilisation, HA functionalisation, and plasma-based release profiling in generating formulations with potential for testing in preclinical animal models of OA. The system’s ability to maintain cell viability while preserving DEX bioactivity strengthens its translational promise, particularly for local therapies that aim to reduce the systemic burden of oral or repeated intra-articular corticosteroid injections. Collectively, these findings position HA-DXP-SLNs as a potential advance in nanomedicine-based approaches for IA administration to treat knee OA. Our next studies will focus on *in vivo* evaluation using clinically relevant animal models that replicate both cartilage and subchondral bone pathology of OA.

## Data Availability

The datasets presented in this study can be found in online repositories. The names of the repository/repositories and accession number(s) can be found below: https://zenodo.org/records/18315340, 1831580; https://zenodo.org/records/18315697, 18315697; https://zenodo.org/records/18315530, 18315530.
